# Supplementary figures and images for: CXCL10-based gene cluster model serves as a potential diagnostic biomarker for premature ovarian failure
Source: PeerJ. 2023 Dec 13;11:e16659. doi: 10.7717/peerj.16659 (PMC10725173; doi:10.7717/peerj.16659)

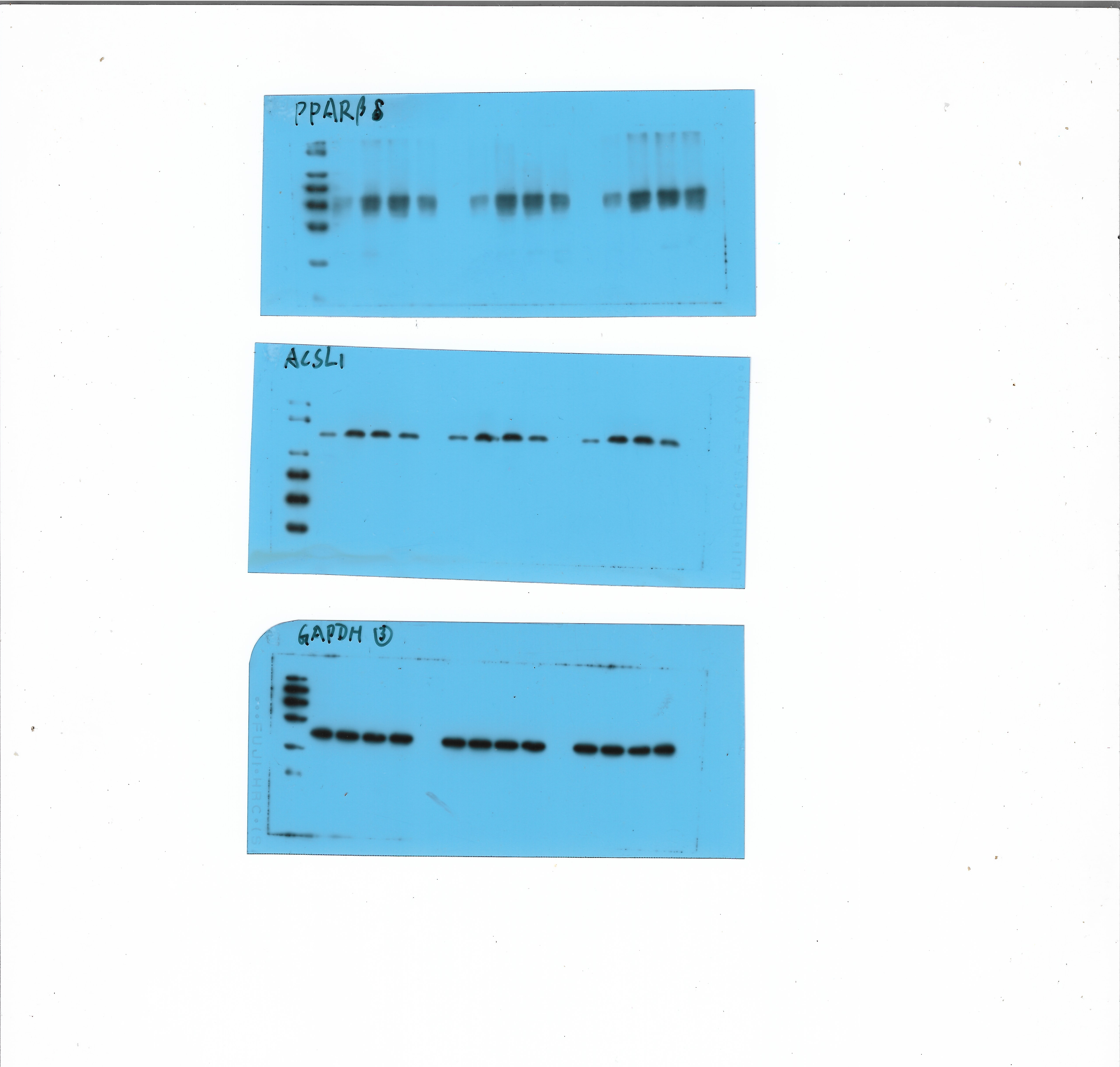

Supplement: Supplemental Information 1 [file peerj-11-16659-s001.zip › Raw data/uncropped wb blots/6C.tif]

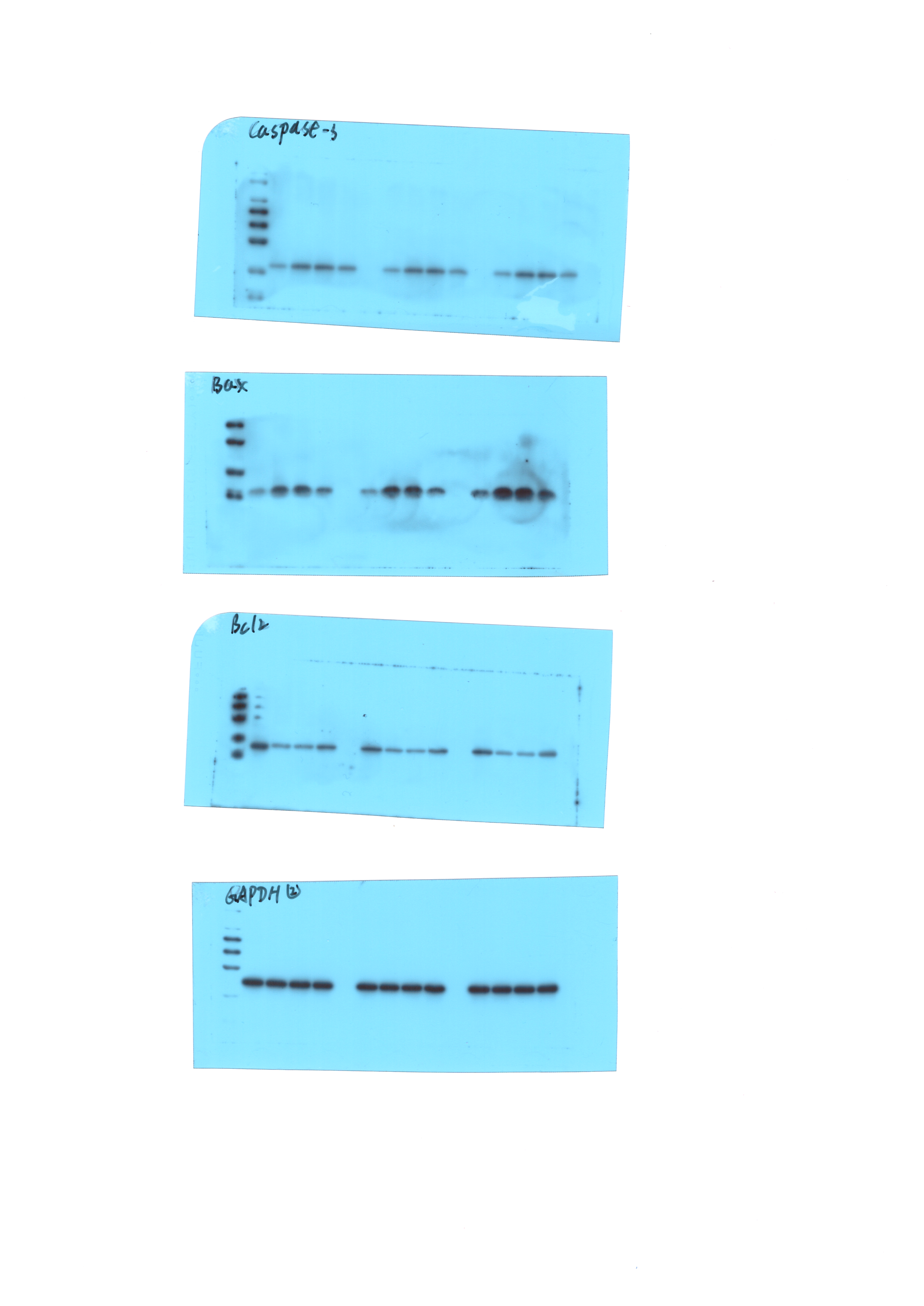

Supplement: Supplemental Information 1 [file peerj-11-16659-s001.zip › Raw data/uncropped wb blots/6B.tif]

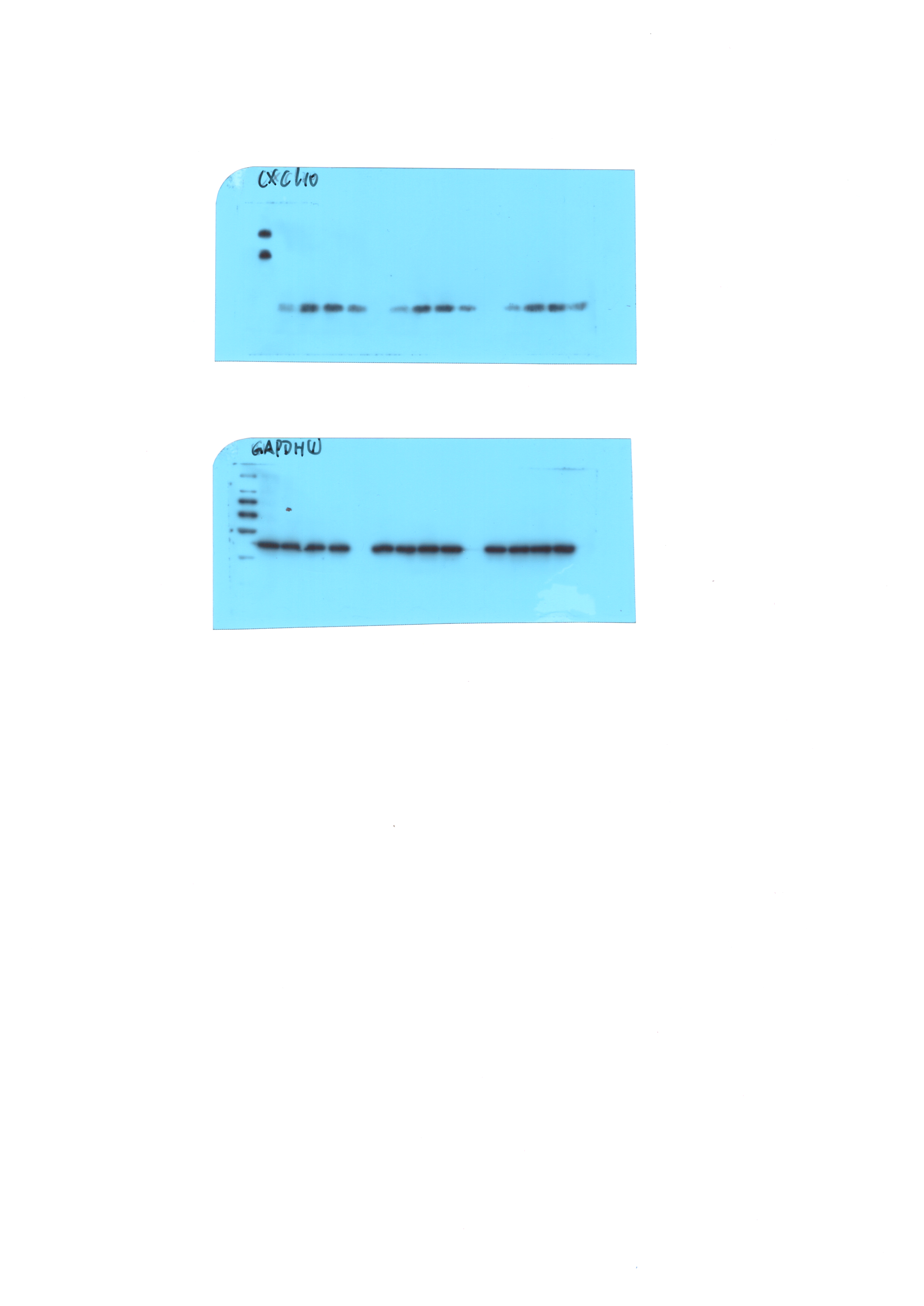

Supplement: Supplemental Information 1 [file peerj-11-16659-s001.zip › Raw data/uncropped wb blots/5D.tif]

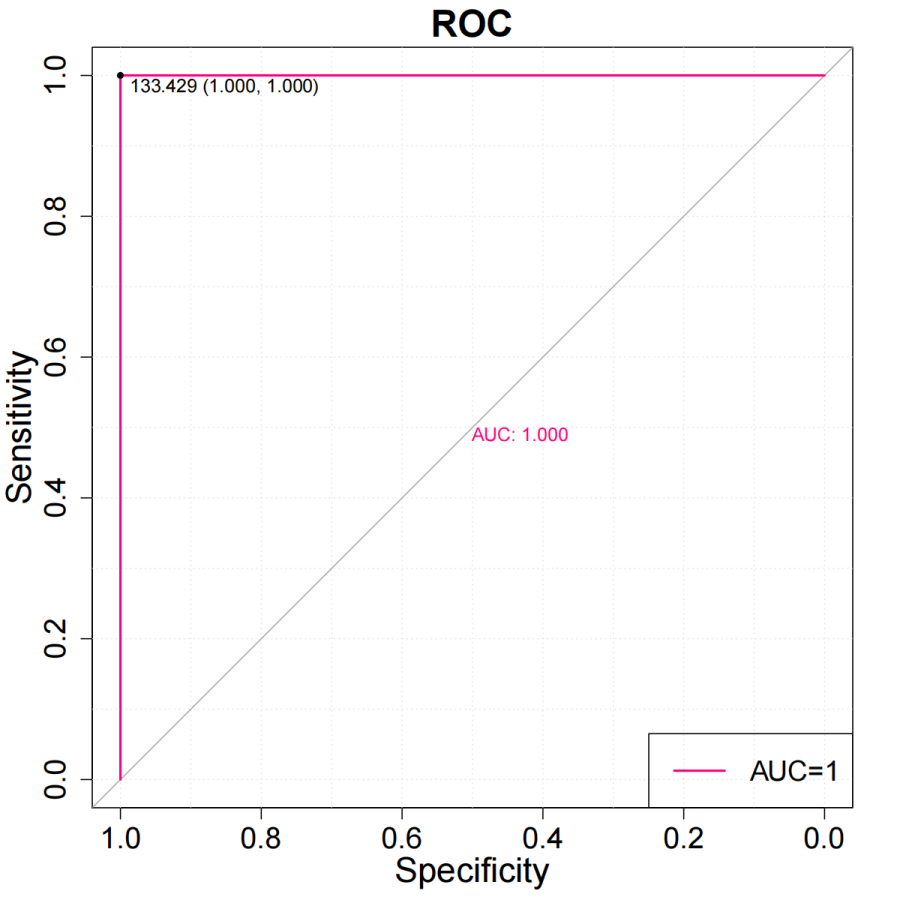


**Figure S1.** The ROC curve showed the diagnosis of Cxcl10-based model genes.

Supplement: Supplemental Information 2 [file peerj-11-16659-s002.zip › Supplemental Files/Supplemental Figures.docx]
