# Supplementary material for: CXCL10-based gene cluster model serves as a potential diagnostic biomarker for premature ovarian failure
Source: PeerJ. 2023 Dec 13;11:e16659. doi: 10.7717/peerj.16659 (PMC10725173; doi:10.7717/peerj.16659)
Supplement: Supplemental Information 3 [file peerj-11-16659-s003.pdf]

ITEM TO CHECK	IMPORTANCE	CHECKLIST
**EXPERIMENTAL DESIGN**		
Definition of experimental and control groups	**E**	Line 93-222
Number within each group	**E**	Line 93-222
Assay carried out by core lab or investigator's lab?	D	N
Acknowledgement of authors' contributions	D	N
**SAMPLE**		
Description	**E**	Line96-98，146-155
Volume/mass of sample processed	D	N
Microdissection or macrodissection	**E**	Line96-98，146-155
Processing procedure	**E**	Line96-98，146-155
If frozen - how and how quickly?	**E**	Line96-98，146-155
If fixed - with what, how quickly?	**E**	Line96-98，146-155
Sample storage conditions and duration (especially for FFPE samples)	**E**	Line96-98，146-155
**NUCLEIC ACID EXTRACTION**		
Procedure and/or instrumentation	**E**	Line 159-166
Name of kit and details of any modifications	**E**	Line 159-166
Source of additional reagents used	D	N
Details of DNase or RNAse treatment	**E**	Line 160-161
Contamination assessment (DNA or RNA)	**E**	Line 161-162
Nucleic acid quantification	**E**	Line 162-163
Instrument and method	**E**	Line 162-164
Purity (A260/A280)	D	Line 163
Yield	D	N
RNA integrity method/instrument	**E**	Line 163-164
RIN/RQI or Cq of 3' and 5' transcripts	**E**	Line 164
Electrophoresis traces	D	N
Inhibition testing (Cq dilutions, spike or other)	**E**	Line 165-166
**REVERSE TRANSCRIPTION**		
Complete reaction conditions	**E**	Line 166-172
Amount of RNA and reaction volume	**E**	Line 166
Priming oligonucleotide (if using GSP) and concentration	**E**	Line167
Reverse transcriptase and concentration	**E**	Line 169
Temperature and time	**E**	Line 166-172
Manufacturer of reagents and catalogue numbers	D	Line 166-172
Cqs with and without RT	D*	N
Storage conditions of cDNA	D	N
**qPCR TARGET INFORMATION**		
If multiplex, efficiency and LOD of each assay.	**E**	Line 181
Sequence accession number	**E**	Line182-183
Location of amplicon	D	N
Amplicon length	**E**	Line187
In silico specificity screen (BLAST, etc)	**E**	Line 188
Pseudogenes, retropseudogenes or other homologs?	D	N
Sequence alignment	D	Line 188
Secondary structure analysis of amplicon	D	N
Location of each primer by exon or intron (if applicable)	**E**	Line 187
What splice variants are targeted?	**E**	Line 182-183
**qPCR OLIGONUCLEOTIDES**		
Primer sequences	**E**	Line 185-186
RTPrimerDB Identification Number	D	N
Probe sequences	D**	N
Location and identity of any modifications	**E**	Line 187
Manufacturer of oligonucleotides	D	N
Purification method	D	N
**qPCR PROTOCOL**		
Complete reaction conditions	**E**	Line172-184
Reaction volume and amount of cDNA/DNA	**E**	Line173
Primer, (probe), Mg++ and dNTP concentrations	**E**	Line 174-175
Polymerase identity and concentration	**E**	Line 175
Buffer/kit identity and manufacturer	**E**	Line174
Exact chemical constitution of the buffer	D	N
Additives (SYBR Green I, DMSO, etc.)	**E**	Line 174
Manufacturer of plates/tubes and catalog number	D	N
Complete thermocycling parameters	**E**	Line176
Reaction setup (manual/robotic)	D	Line175-177
Manufacturer of qPCR instrument	**E**	Line 172-173
**qPCR VALIDATION**		
Evidence of optimisation (from gradients)	D	N
Specificity (gel, sequence, melt, or digest)	**E**	Line 188-189
For SYBR Green I, Cq of the NTC	**E**	Line 189-190
Standard curves with slope and y-intercept	**E**	Line 178-179
PCR efficiency calculated from slope	**E**	Line 177-181

Confidence interval for PCR efficiency or standard error	D	Line 181
r2 of standard curve	**E**	Line 179
Linear dynamic range	**E**	Line 179
Cq variation at lower limit	**E**	Line 179
Confidence intervals throughout range	D	Line 181
Evidence for limit of detection	**E**	Line179-181
If multiplex, efficiency and LOD of each assay.	**E**	Line 181
**DATA ANALYSIS**		
qPCR analysis program (source, version)	**E**	Line183-184
Cq method determination	**E**	Line181-182
Outlier identification and disposition	**E**	Line190-191
Results of NTCs	**E**	Line 190
Justification of number and choice of reference genes	**E**	Line 184
Description of normalisation method	**E**	Line181-183
Number and concordance of biological replicates	D	N
Number and stage (RT or qPCR) of technical replicates	**E**	Line 192-193
Repeatability (intra-assay variation)	E	Line 190-193
Reproducibility (inter-assay variation, %CV)	D	N
Power analysis	D	N
Statistical methods for result significance	**E**	Line 221-222
Software (source, version)	E	Line 216
Cq or raw data submission using RDML	**D**	N

Table 1. MIQE checklist for authors, reviewers and editors. All essential information (E) must be submitted with the manuscript. Desirable information (D) should be submitted if available. If using primers obtained from RTPrimerDB, information on qPCR target, oligonucleotides, protocols and validation is available from that source.

*: Assessing the absence of DNA using a no RT assay is essential when first extracting RNA. Once the sample has been validated as RDNA-free, inclusion of a no-RT control is desirable, but no longer essential.

**: Disclosure of the probe sequence is highly desirable and strongly encouraged. However, since not all commercial pre-designed assay vendors provide this information, it cannot be an essential requirement. Use of such assays is advised against.
